# Supplementary material for: The causes of loneliness: The perspective of young adults in London’s most deprived areas
Source: PLoS One. 2022 Apr 6;17(4):e0264638. doi: 10.1371/journal.pone.0264638 (PMC8985970; doi:10.1371/journal.pone.0264638)
Supplement: Attachment — Submitted filename: Response to Reviewers.docx [file pone.0264638.s001.docx]

Dear Editor and Reviewers,

Thank you so much for the very useful comments and suggestions for change. We have addressed each, in turn, below (please see the reviewer’s remarks in bold and in “” and our responses underneath each point):

**“Please ensure that your manuscript meets PLOS ONE's style requirements, including those for file naming.”**

We have tried to achieve this, and any changes are marked with yellow highlights in the ‘Revised Manuscript with Track Changes’ document.

**“Please note that funding information should not appear in the Acknowledgments section or other areas of your manuscript. We will only publish funding information present in the Funding Statement section of the online submission form.”**

We have removed funding information from the Acknowledgements. Please see the Acknowledgements. Please also see the Cover Letter for a revised Funding Statement.

**“We note that you have indicated that data from this study are available upon request.”**

Please see the Cover Letter for this.

**“Please review your reference list to ensure that it is complete and correct. If you have cited papers that have been retracted, please include the rationale for doing so in the manuscript text or remove these references and replace them with relevant current references.”**

We covered reference number 38 with black because it is a paper we recently published. We decided to keep it blind until the review process is complete. This note is written above this reference and highlighted in yellow in the References section.

**Reviewer 1**

**“It would be better to define a technique for sampling. The authors did not mention about the technique to identify the deprived areas of London. There is need to enhance the sample size of the study as well. Qualitatively, authors proved the results with regard to hypothesis.”**

We have now stated that this was a purposive sample. We have also added a new paragraph called ‘Neighbourhood selection’ to demonstrate the technique we used to identify the deprived areas. Please note the reference number 57 both in the same paragraph and in the paragraph about the participants, where the source for this identification was obtained.

As reviewer 1 states, this is a qualitative study, and the sample size is sufficient for the research questions asked. Of course, a quantitative study would require larger numbers, and so we have said (lines 671-674) that were one to extend this study with a quantitative approach, one would have to have a larger sample size determined by way of a power analysis.

**Reviewer 2**

1. **“Line 194-195: The first research question is a general one (the subjective causes). The authors added the second question (the role of social media). When this is written as one of the aim, I would expect that the role of social media will be explored via a specific question during the interview. When it was not the case, authors mighty consider to exclude this research question.”**

We have left this question in for two reasons. Firstly, in the method used for this paper, no specific questions are used. One asks people to free associate, in this case about the experience of loneliness and then explores what they say. Based on the existing literature we were interested in what they saw as the cause/s of loneliness and, since the social science literature increasingly links social media use to loneliness/illbeing, it felt important to explore whether this facet would feature as a cause of loneliness. Secondly, we have left in this specific focus because in this field a lot of the work is highly reductionist stating whether the social media is good or bad for young people depending on how much they use it. The area lacks subtlety and theory. We endeavour to introduce, in this exploratory work, subtlety and relevant theory.

2. **“Table 1: For consistency, also add percentage for each boroughs in the 'Total' column.”**

Percentages for each borough in the ‘Total’ column are added.

3. **“Line 253 - 265 - Data Analysis: Please elaborate the steps of data analysis being done: how many coders work independently? When was the second coder did the double coded to ensure reliability? Any improvement or changes being done after this comparison? Etc.”**

Lines 267-279 explain the steps. We have added that the codes were inductive and that the two coders worked independently. In addition, we have added what was requested in relation to when the double-coding was conducted, and improvements/changes made after the comparison.

4. **“Line 622-626: It is stated that responses were compared across gender, race and religion. Authors mentioned the females, BAME and no religion more likely to socially compare themselves. However, I could not find description in the Results section (theme 4: social comparison) regarding comparison between race and religions, only between gender. Please elaborate.”**

Thanks for identifying this omission. ‘Race’ and ‘religion’ regarding comparison are now added under the theme of ‘social comparison’ within the Results section.

5. **“Line 646-647 - Limitation: I would argue that this is not a limitation, since the research question and aim of the present study was to explore loneliness in the young adults from deprived area, no comparison was intended.”**

Thank you. We have removed reference to a limitation. We have, however, left in some of the contents of the paragraph to indicate that a future study that included those from wealthier boroughs would allow us to compare causes of loneliness in wealthier versus more deprived young adults.
